# Using machine learning to predict the small for gestational age and identify the important predictors: A real-world clinical cohort study in China

**DOI:** 10.1371/journal.pone.0343994

**Published:** 2026-03-25

**Authors:** Yimin Zhang, Zheng Liu, Jingyao Liu, Jing Chen, Xiaorui Zhang

**Affiliations:** 1 Department of Pediatrics, Peking University People’s Hospital, Beijing, China; 2 Department of Maternal and Child Health, School of Public Health, Peking University, Beijing, China; KI: Karolinska Institutet, SWEDEN

## Abstract

**Purpose:**

Aims to use machine learning to predict the risk of small for gestational age (SGA) and identify its important predictors.

**Methods:**

This is a retrospective cohort study conducted from December 20, 2023, to May 20, 2024, focusing on newborns and their mothers who delivered at Peking University People’s Hospital from January 1, 2012, to December 31, 2022. We included a total of 18,164 pregnant women. We adopted 7 machine-learning-based models (2 linear models, 4 tree-based models, and 1 ensemble learning model).

**Results:**

Altogether, 1437 (7.9%) pregnant women delivered SGA births. Among them, 27.7% and 72.3% were moderate-to-severe and mild types of SGA, respectively, and the percentages of term and preterm SGA were 88.1% and 11.9%, respectively. Although the ridge classifier (linear-based model) performed better than the other 6 models in terms of model discrimination (AUROC: 0.71), the performance of all 7 models in calibration remained unsatisfactory. All of them tended to underestimate the risk of SGA and could not capture approximately half of the SGA births (recall: 0.49). Maternal height was shown as the most important predictor for the SGA, moderate-to-severe SGA, full-term SGA, and preterm SGA, even outweighing the predictors of pre-pregnancy BMI and gestational weight gain. For mothers shorter than 158 cm, their risk of delivering SGA births was 3.61 (95% CI: 2.91 to 4.50) per 1-SD decrease in height, but for those higher than 158 cm, the SGA risk was shown no evidence of association with maternal height (*P* > 0.05).

**Conclusions:**

Our study not only contributes a basic model for the prediction of SGA, but also identified the short maternal height as a previously neglected predictor of SGA.

## Introduction

Globally, the small for gestational age (SGA) is estimated to influence 32 million newborns annually [1,2], with incidence rates varying across countries, ranging from 6.7% to 12.9% [2,3]. SGA newborns are vulnerable to lifelong loss of human capital due to their increased risk of morbidity and mortality, delay in physical growth and development, and dysfunction in cognition and neurodevelopment in the whole life circle [4–8]. This public health and clinical health concern brings a heavy burden for individuals, families, and the whole society, which is more pressing in low- and middle-income countries [9].

Timely identification of the high-risk pregnancy group of SGA at early pregnancy or even before pregnancy is highly important. This could enable clinicians to take preventative and treatment steps and offer intensive monitoring to this vulnerable group during postnatal care. Currently, detection of SGA primarily relies on (1) ultrasound measurements of fetal growth, and (2) maternal serum biomarkers [10]. Firstly, the routine ultrasound to evaluate fetal growth was estimated to miss 20%−50% of diagnoses [11], and access to resource-dependent ultrasound use is not fully available for every pregnant woman in low- and middle-income countries [12]. Secondly, while the measurements of maternal serum biomarkers hold great potential to improve the sensitivity of diagnosing SGA, given its costs, invasiveness, and under-coverage in routine prenatal care up to now, there remains a critical need for the prediction of SGA based on the easy-available, real-world clinical data.

The multifactorial nature of SGA presents a significant challenge to developing a valid clinical model for its accurate prediction. The logistic regression model has been commonly used to classify SGA and non-SGA neonates [13]. However, this model has strict assumptions under its mathematical algorithms of the regression coefficients. One important assumption is the independence of the predictors, which might often be violated in the actual prediction of SGA; for example, pregnancies with gestational diabetes are more likely to develop gestational hypertension or other pregnancy comorbidities (that is, predictors of SGA are often correlated and not independent from each other). Another assumption is that the associations between predictors and the outcome are linear, but it can also be violated in fact; for example, the association of maternal height with SGA risk might exert a different pattern between those with short stature and those with high stature. As such, more flexible prediction models are urgently needed to complement the limitations of logistic regression for complex prediction tasks like SGA prediction.

In addition to the development of a prediction model for SGA, it is also clinically important to interpret how much the predictor contributes to the prediction of the outcome, which is also called model interpretability [14]. This can greatly facilitate the clinicians to bridge the wide gap of translating the research findings into clinical practice. Furthermore, the identification of a limited set of important predictors could help to build a brief and practical model, which is critically important in generalizing the research findings to the population-wide, real-world settings. For example, in the current management of high-risk pregnancies in China, stratification of care for varying risks of pregnancies is mainly based on one specific risk factor [15].

Based on our systematic review of this topic, previous studies have predominantly focused on a single type of SGA. For instance, a Finland study [16] demonstrated that preterm infants with SGA are associated with a broad spectrum of neurodevelopmental and psychiatric disorders. However, few studies have examined the various specific subtypes of SGA, including moderate to severe SGA, term SGA, and preterm SGA. The risks of mortality, growth and development delay, neurodevelopmental disorders, and various perinatal diseases in children were shown to increase with the extent of severity of SGA [17,18]. Regarding the term SGA, its timely diagnosis is often overlooked until the physical examinations in the childhood period, but the perinatal and long-term prognosis of term SGA is poorer than the appropriate-for-gestational-age (AGA) infants born at term [19]. Additionally, the established models of predicting the risk of delivering an SGA neonate have been limited in the variety of predictors, insufficient sample size [20], and generalizability to low- and middle-income countries [21]. Fortunately, previous cross-sectional studies have indicated a slight downward trend in the incidence of SGA in China [21,22]. This may be attributed to an increased proportion of mothers accessing high-quality healthcare [23]; however, the specific reasons remain unclear, highlighting the critical need for a systematic predictive model for SGA occurrence. Consequently, this study utilized a 10-year cohort in China, which collected clinically validated, accessible real-world predictors within clinical settings. The research aims to first compare the performance of multiple machine learning models in predicting SGA risk, and then investigate whether the ranking of predictor importance varies across specific SGA (moderate-to-severe SGA, term SGA, preterm SGA).

## Methods

We followed the guideline of Transparent Reporting of a Multivariable Prediction Model for Individual Prognosis or Diagnosis (TRIPOD) [24,25]. All methods were performed in accordance with the relevant guidelines and regulations.

### Study population

This is a retrospective cohort study conducted from 20/12/2023 to 20/05/2024, focusing on newborns and their mothers who delivered at Peking University People’s Hospital from 01/01/2012 to 31/12/2022. We included pregnant women who received usual prenatal care and gave birth in this hospital and excluded those with potentially implausible measurements of maternal height, weight, or newborns’ length, weight, or gestational age. This study was performed in accordance with the Declaration of Helsinki and was approved by the local ethics review board (2023PHB353). Informed consent was obtained from all subjects/patients.

### Measures and outcomes of SGA

The primary outcome of this study was the presence of the general type of SGA, defined as birth weight less than the 10^th^ percentile of sex- and gestational age-specific reference values [6]. The secondary outcomes included the two specific types of SGA: (1) moderate-to-severe SGA, defined as birth weight less than the 5^th^ percentile of sex- and gestational age-specific reference values (the complementary type was the mild SGA, defined as birth weight ranging from 5^th^ to 10^th^ percentile of corresponding values) [6]; (2) term SGA, defined as the delivery of SGA not earlier than 37 weeks of gestation (the complementary type was the preterm SGA, defined as the delivery of SGA earlier than 37 weeks of gestation). To ensure the robustness of the study results, we carefully validated the measures of birth weight, gestational week, and the diagnosis of general or specific types of SGA by at least two researchers in our study team who had 20 + years of experience in clinical practice and data preprocessing.

### Predictors of SGA

We selected the predictors of SGA mainly based on two considerations: first, measures of the SGA predictors had been retrospectively collected from the routine clinical care for the vast majority of the general pregnant women; second, the SGA predictors had been indicated to be linked to the risk of SGA due to the underlying biological mechanisms based on the literature review [26]. The first consideration was aimed to ensure data availability of the study and thus its findings could be feasibly generalized to the real-world settings. The second consideration was aimed to echo the call to establish the causal association between the predictors and the outcome instead of the sole statistical association, and achieving this end, the established model might have a better ability to interpret the outcome and also be easier accepted by the clinicians.

The predictors selected for this study included those collected in pre-pregnancy health care and those collected in prenatal care. The first type of predictors included maternal height, pre-pregnancy body mass index (BMI), age, parity, and the presence or absence of assisted reproduction. The second type of predictors included gestational weight gain and the presence or absence of pregnancy-related complications (multiple gestation, anemia, diabetes, hypertension, preeclampsia, hyperthyroidism, hypothyroidism, thyroid cancer, connective tissue disease, cord around neck, intrauterine growth restriction, battledore placenta, and velamentous placenta).

### Establishment of machine-learning-based models

We adopted seven classification models to predict SGA status, including two linear models (ridge regression and logistic regression), four tree-based models (random forest, XGBoost, CatBoost, and LightGBM), and one ensemble model. The ensemble model, which integrated predictions from all the aforementioned linear and tree-based models, was specifically utilized to mitigate potential performance issues arising from class imbalance in the dataset.

This selection of seven models represented a spectrum of model complexity and interpretability [14], ranging from simple, interpretable linear models to the complex, less interpretable ensemble, with tree-based models falling in between. Our final model was chosen by comprehensively balancing model performance, complexity, and model interpretability, adhering to the currently proposed guidelines for the routine use of machine learning algorithms by the European Commission [27,28] and the WHO [29]. We stepwise established the machine-learning-based models through model training, hyperparameter tuning, and 5-fold cross-validation. We used the scikit-learn [30] 1.4.2 in Python 3.11.7 for machine learning.

### Evaluation of model performance

We assessed the model performance in discrimination and calibration. Specifically, discrimination refers to the ability of the developed model to distinguish between individuals with the outcome and those without the outcome, while calibration refers to the extent of consistency between the actual outcomes and predicted outcomes. To evaluate discrimination, we used measures of both the Area Under the Receiver Operating Characteristics curve (AUROC) and that of the Precision-Recall curve (AUPRC, also called average precision, the calculated formula 1 shown below), as the AUPRC has been acknowledged as the necessary indicator complimentary to the former in the case of the unbalanced data as in this study [31]. We also showed other evaluation indicators of discrimination including precision, recall, and F1 score (a harmonic means of the precision and recall, the calculated formula 2 shown below). To evaluate calibration intuitively, we plotted the calibration curve showing the predicted risks of outcome on the x-axis and observed outcome on the y-axis.

- Formula 1:$AUPRC=\sum_{\;n}({Recall}_{n}-{Recall}_{n-\text{1}}){Precision}_{n}$

(${Precision}_{n}$ and ${Recall}_{n}$ are the precision and recall at the n^th^ threshold.)

- Formula 2: $F\text{1}=\;\frac{\text{2}\times TP}{\text{2}\times TP+FP+FN}$

(*TP*: the number of true positives; *FN*: the number of false negatives; *FP*: the number of false positives.)

### Interpretation of model prediction results

We interpreted the model prediction results in two steps. First, we evaluated the extent of the model dependent on the predictors, and the greater the extent, the more important the predictor. Second, we examined the direction and shape of the association between the predictor (continuous variable) and the outcome; that is, whether the risks of the outcome increased with the increase of the predictor or the case was the opposite, and also, whether the predictor-outcome associations were linear or curvilinear. For linear models, we used the standardized regression coefficients to conduct the above steps, while for tree-based models and ensemble learning, we used the SHAP (SHapley Additive exPlanations) values [32].

## Results

### Characteristics of the study population

This study included a total of 18,164 mother-offspring dyads, 1437 (7.9%) of them were diagnosed as SGA. The SGA neonates were first categorized according to the extent of severity and 398 (27.7%) of them were moderate-to-severe type of SGA while the remaining were the mild type. Then, the SGA neonates were classified in terms of the gestational age and 1266 (88.1%) and 171 (11.9%) of them were term SGA and preterm SGA, respectively. Table 1 and 2 compares the differences in characteristics of non-SGA, mild SGA, and moderate-to-severe SGA, and those of non-SGA, term SGA, and preterm SGA, respectively.

**Table 1:** Characteristics of the study population (categorized into non-SGA, mild SGA, and moderate-to-severe SGA).

	Non-SGA (N = 16,727)	Mild SGA (N = 1039)	Moderate-to-severe SGA (N = 398)	*P*
**Continuous variables, mean (SD)**				
Gestational week, week	38.30 (1.88)	38.27 (2.21)	38.28 (1.94)	0.845
Birth weight, g	3237.63 (540.13)	2599.33 (434.76)	2248.73 (416.58)	<0.001
Maternal height, cm	162.79 (4.73)	159.79 (5.76)	160.43 (6.45)	<0.001
Gestational weight gain, kg	13.97 (4.74)	13.21 (4.45)	13.63 (4.96)	<0.001
Pre-pregnancy BMI, kg/m^2^	23.30 (3.85)	22.87 (3.72)	23.47 (4.16)	0.001
Maternal age, year	31.82 (4.14)	31.87 (4.23)	31.42 (3.78)	0.113
**Categorical variables, n (%)**				
Assisted reproduction	25 (0.1)	11 (1.1)	7 (1.8)	<0.001
Primi-gravidity	13191 (79.6)	882 (85.8)	332 (84.3)	<0.001
Primiparity	15466 (92.5)	993 (95.6)	373 (93.7)	0.001
Female newborn	8144 (48.7)	330 (31.8)	94 (23.6)	<0.001
Gemellary pregnancy	741 (4.4)	77 (7.4)	53 (13.3)	<0.001
Intrauterine growth restriction	305 (1.8)	121 (11.6)	80 (20.1)	<0.001
Hypertension	1711 (10.2)	133 (12.8)	69 (17.3)	<0.001
Preeclampsia	911 (5.4)	105 (10.1)	42 (10.6)	<0.001
Diabetes	3251 (19.4)	176 (16.9)	85 (21.4)	0.083
Anemia	6250 (37.4)	355 (34.2)	151 (37.9)	0.112
Desmosis	331 (2.0)	40 (3.8)	28 (7.0)	<0.001
Cord around neck	6713 (40.1)	460 (44.3)	151 (37.9)	0.019
Lobstein’s placenta	271 (1.6)	31 (3.0)	20 (5.0)	<0.001
Battledore placenta	20 (0.1)	9 (0.9)	9 (2.3)	<0.001
Hyperthyroidism	260 (1.6)	8 (0.8)	1 (0.3)	0.015
Hypothyroidism	2139 (12.8)	132 (12.7)	62 (15.6)	0.256
Thyroid cancer	46 (0.3)	1 (0.1)	0 (0.0)	0.588

Abbreviations: SGA = small for gestational age; SD = standard deviation; BMI = body mass index.

**Table 2:** Characteristics of the study population (categorized into non-SGA, term SGA, and preterm SGA).

	Non-SGA (N = 16,727)	Term SGA (N = 1266)	Preterm SG (N = 171)	*P*
**Continuous variables, mean (SD)**				
Gestational week, week	38.30 (1.88)	38.89 (1.05)	33.71 (2.56)	<0.001
Birth weight, g	3237.63 (540.13)	2626.29 (283.57)	1583.74 (453.76)	<0.001
Maternal height, cm	162.79 (4.73)	159.95 (5.98)	160.09 (5.84)	<0.001
Gestational weight gain, kg	13.97 (4.74)	13.29 (4.52)	13.63 (5.14)	<0.001
Pre-pregnancy BMI, kg/m^2^	23.30 (3.85)	23.02 (3.87)	23.17 (3.79)	0.044
Maternal age, year	31.82 (4.14)	31.73 (4.14)	31.84 (3.92)	0.774
**Categorical variables, n (%)**				
Assisted reproduction	25 (0.1)	6 (0.5)	12 (7.0)	<0.001
Primi-gravidity	13191 (79.6)	1097 (86.7)	117 (75.0)	<0.001
Primiparity	15466 (92.5)	1216 (96.1)	150 (87.7)	<0.001
Female newborn	8144 (48.7)	363 (28.7)	61 (35.7)	<0.001
Gemellary pregnancy	741 (4.4)	79 (6.2)	51 (29.8)	<0.001
Intrauterine growth restriction	305 (1.8)	131 (10.3)	70 (40.9)	<0.001
Hypertension	1711 (10.2)	150 (11.8)	52 (30.4)	<0.001
Preeclampsia	911 (5.4)	89 (7.0)	58 (33.9)	<0.001
Diabetes	3251 (19.4)	213 (16.8)	48 (28.1)	0.001
Anemia	6250 (37.4)	431 (34.0)	75 (43.9)	0.012
Desmosis	331 (2.0)	46 (3.6)	22 (12.9)	<0.001
Cord around neck	6713 (40.1)	555 (43.8)	56 (32.7)	0.004
Lobstein’s placenta	271 (1.6)	37 (2.9)	14 (8.2)	<0.001
Battledore placenta	20 (0.1)	12 (0.9)	6 (3.5)	<0.001
Hyperthyroidism	260 (1.6)	7 (0.6)	2 (1.2)	0.007
Hypothyroidism	2139 (12.8)	170 (13.4)	24 (14.0)	0.723
Thyroid cancer	46 (0.3)	1 (0.1)	0 (0.0)	0.524

Abbreviations: SGA = small for gestational age; SD = standard deviation; BMI = body mass index.

### Model performance in the prediction of SGA

**Fig 1:**
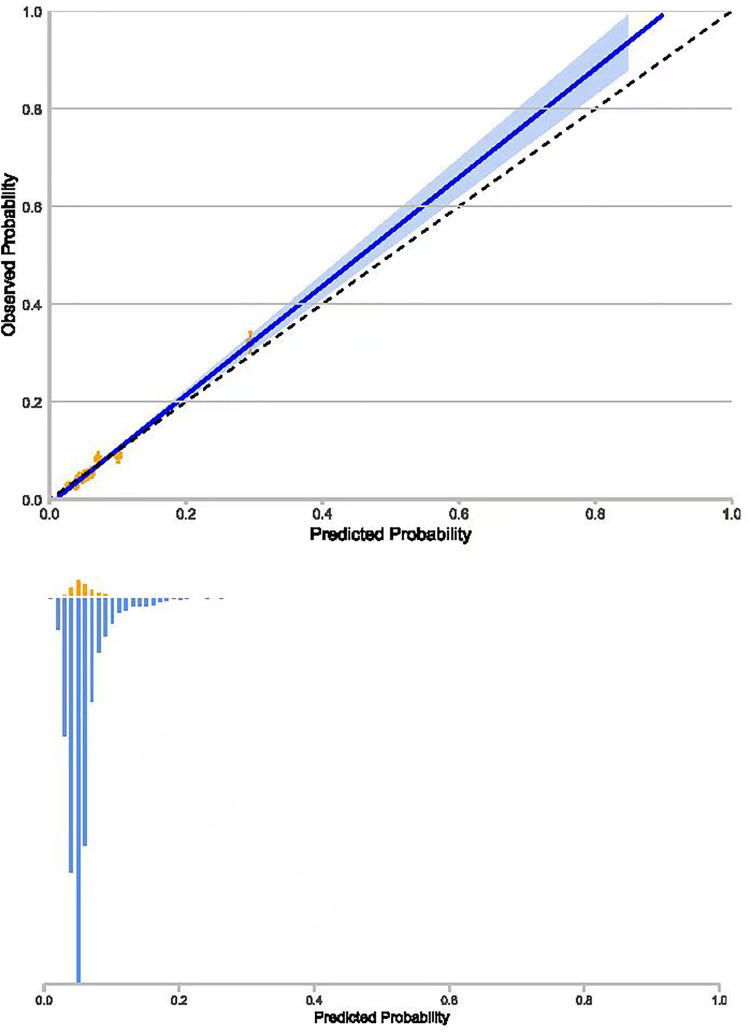
Calibration plot for the prediction of SGA. (The area above the dashed line indicates that the model’s risk estimates are low; 10 orange circles indicate the 10 groups defined by a tenth of the distribution of estimated risks; histograms at the bottom of graphs show the distribution of the estimated probability of SGA in the group of SGA (orange colour) and that in the group of non-SGA newborns (blue colour)).

The discrimination performance of the 7 prediction models in AUROC, AUPRC, precision, recall, and F1 score is shown in Table 3. The linear model of the ridge classifier performed better than the other 6 models in terms of AUROC, AUPRC, and F1 score (Table 3). As for the calibration performance (Fig. 1), the predicted risks derived from the models were largely consistent with the actual risk among the low-risk (estimated probability < 0.1) population, but the models tended to under-estimate the risk of SGA among the high-risk (estimated probability > 0.1) population.

**Table 3:** Discrimination performance of the seven prediction models in predicting the SGA.

Models	AUROC	AUPRC	Precision	Recall	F1 score
*Linear models*					
Ridge classifier	0.71	0.28	0.25	0.49	0.32
Logistic regression	0.69	0.27	0.17	0.57	0.26
*Tree-based models*					
Random forest	0.65	0.16	0.18	0.36	0.24
XGBoost	0.65	0.18	0.23	0.31	0.26
CatBoost	0.67	0.21	0.27	0.35	0.29
LightGBM	0.67	0.21	0.26	0.35	0.30
*Ensemble learning*					
Ensemble	0.70	0.26	0.28	0.39	0.31

Abbreviations: AUROC = the Area Under the Receiver Operating Characteristics curve; AUPRC = the Area Under the Precision-Recall curve

We also plotted a nomogram to interpret how the model could be applied in practice (see Supplemental Fig 1). As an illustrative example, consider a pregnant woman aged 31 years with a maternal height of 163 cm, pre-pregnancy BMI of 22.7 kg/m², and gestational weight gain of 14.0 kg. She was primigravid and primiparous. She had anemia, hypothyroidism, Lobstein placenta, and intrauterine growth retardation, but no gemellary pregnancy, hypertension, preeclampsia, diabetes, hyperthyroidism, thyroid cancer, systemic lupus erythematosus, or cord around neck. In the nomogram, each predictor value is located on its corresponding axis and projected to the points (0–100) scale; the resulting scores are summed to obtain total points, which are then converted to the predicted probability of SGA on the probability scale. For this example, the total points were 189.02, corresponding to a predicted probability of SGA of 2.35%.

### Important predictors for the risk of SGA

**Fig 2:**
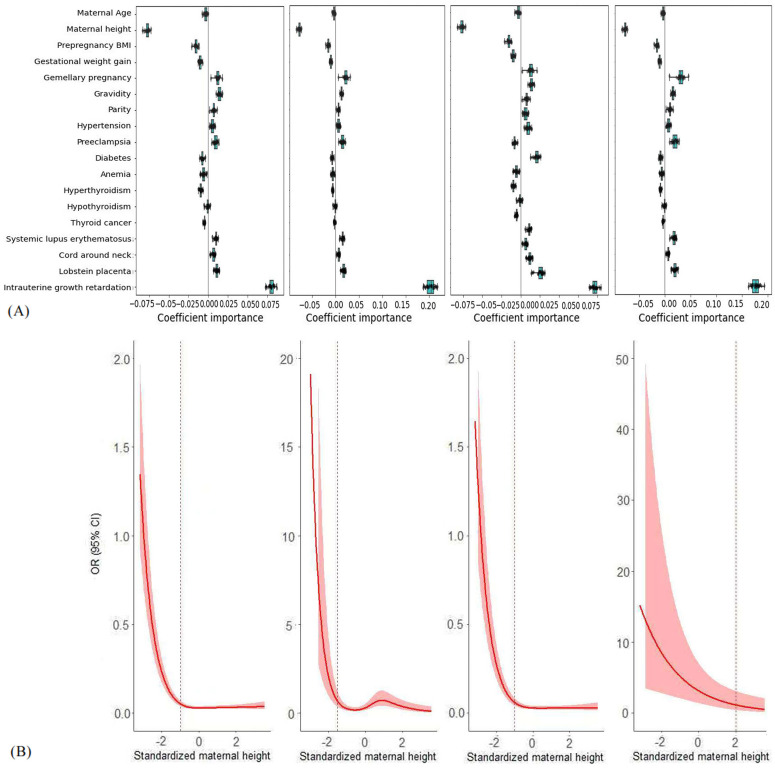
Evaluation of predictor importance for the risk of SGA. (The top half: the boxplots of standardized coefficients of predictors in the ridge regression model during 5-fold cross-validation repeated 5 times, and the finding indicates that the farther away from the vertical line, the more important the predictor; the bottom half: the adjusted OR (95% CI) of SGA along with the change of the standardized maternal height in the restricted cubic regression model. From left to right, the outcomes in the model were the general type of SGA among the whole population, moderate-to-severe SGA among the neonates with SGA, full-term SGA among the neonates born at term, and preterm SGA among the neonates born preterm).

First, we used the ridge classifier to identify important predictors for the risk of SGA. Fig 2(A) compares the standardized regression coefficients in (1) predicting the risk of the general type of SGA among the whole population, (2) predicting the risk of moderate-to-serious SGA among the SGA neonates, (3) predicting the risk of term SGA among the SGA and non-SGA neonates born at term, and (4) predicting the risk of preterm SGA among the SGA and non-SGA neonates born preterm. In all of the 4 prediction tasks, maternal height was shown as the most important predictor that contributed to the prediction results (Fig 2(A)).

Then, we examined the association of maternal height with the risk of SGA in detail. Results from our restricted cubic regression analyses showed that the association of maternal height with the outcome followed a curvilinear shape (Fig 2(B)); that is, when the maternal height was below 158 cm (mean – 1 standard deviation), they had a higher risk of delivering SGA neonates per 1-SD decrease in the maternal stature (OR: 3.61; 95% CI: 2.91–4.50), but in comparison, when the maternal height was above 158 cm, the risk of delivering SGA neonates remained relatively stable with the change of maternal stature.

## Discussion

In low- and middle-income countries, the progress in preventing the delivery of SGA remained slow despite the great efforts in maternal and child care [33]. The primary challenges for China stem from shifts in family planning policies over the past decade—transitioning from one-child to two-child and now multi-child policies—coupled with rising maternal age and high-risk pregnancies. [34,35]. However, recent research based on national Chinese data indicates a declining trend in the occurrence of SGA [22]. To investigate the reasons behind this change, our study takes advantage of a 10-year, clinician-validated, monocenter cohort involving 18,164 mother-offspring dyads in China and builds the interpretable, machine-learning-based prediction model for the risk of SGA. Using only the non-invasive, easy-to-collect predictors, our developed model indicated the potential of model discrimination and calibration, and the interpretation results of the model showed that maternal height, a previously neglected predictor, showed consistency in predicting the general type of SGA, moderate-to-severe SGA, term SGA, and preterm SGA.

The existing studies of predicting the risk of SGA were heterogeneous in mainly 4 aspects: sample size and ethnicity of the study population, number and type of predictors, the complexity of prediction models, and evaluation methods of model performance. Concerning the study population, a systematic review [13] has summarized that most of the previous studies were conducted in high-income countries, but it is in low- and middle-income countries that the burden of neonatal and post-neonatal deaths and diseases is more serious [36], and most of these are preventable through timely identification of SGA. Additionally, the sample size used in more than 78% of the previous studies was less than 10,000. This might not allow for reliable prediction modeling: 1) in the scenario of linear regression models, a common rule of thumb is to require at least 10 events (the occurrence of SGA) per variable for linear regression models [37], and to put it alternatively, the lower prevalence of SGA and the greater number of predictors, the large sample size is needed to ensure the reliability of the prediction modeling; 2) in the scenario of machine-learning-based models, there has not existed the standard method to estimate the minimum sample size, and some evidence suggests that much more data may be required for some machine learning methods [38].

Regarding the predictors, our study included a total of 22 predictors covering the types of maternal nutritional status, uterine and cervical factors, and fetal characteristics. These predictors were easy to collect through a retrospective review of clinical records and can be regarded as a baseline model of clinical prediction for SGA. Another study also stressed the importance of baseline (basic) models as they only included variables that can be assessed non-invasively; in comparison, the extended models additionally included variables such as serum glucose concentration [39]. This study of the systematic review found that prediction results from the extended models were only minimally better than the baseline models in the prediction of type 2 diabetes. However, the strategic integration of evidence-based advanced predictors holds significant potential for enhancing the accuracy of SGA prediction. Future research could focus on three key dimensions: First, placental biomarkers: Incorporating markers of placental dysfunction, such as serum placental growth factor (PlGF), may capture early pathological pathways leading to SGA [40]. The International Federation of Gynecology and Obstetrics states in its Guidelines for Intrauterine Growth Restriction (2021 edition) that mid-pregnancy PlGF levels may be associated with SGA occurrence [41]. Second, dynamic fetal biometry: Serial ultrasound measurements, such as estimated fetal weight（EFW）, can provide real-time growth trajectory data [42]. Machine learning analysis of longitudinal EFW data may identify abnormal growth patterns earlier than single-timepoint assessments. Third, environmental and social risks: Quantifying indoor/environmental pollutants (e.g., PM2.5 via geocoding or wearable sensors), maternal infections (e.g., malaria/ Human Immunodeficiency Virus), and lifestyle factors (diet, stress) can address population-specific risk heterogeneity [26]. To implement these advances, future studies should: Incorporate biomarkers while ensuring privacy protection for multicenter data integration; Conduct cost-effectiveness analyses to evaluate the clinical utility versus resource requirements of expanded models; Although model complexity must be balanced with clinical feasibility, targeted expansion of predictive factors represents a crucial pathway for the precise prevention of SGA.

Nevertheless, we should bear in mind that further collection of the evidence-based predictors has a great potential to boost the model performance in the prediction of SGA, primarily including sonographic measures such as estimated fetal weight [42], biomarkers indicating impaired placentation such as the serum placental growth factor [43], maternal infection with malaria or Human Immunodeficiency Virus, environmental exposures such as ambient and indoor air pollution, and lifestyle during pregnancy [26].

Concerning the prediction models, our study adopted both the conventional linear regression models and the complex machine-learning-based models. After comprehensive consideration of model performance, interpretation, and parsimony, the model of ridge regression outperformed the other models. This might be related to the advantages of ridge regression in addressing the collinearity and overfitting issue of ordinary least squares via imposing a penalty on the size of the coefficients [44]. Our results also suggest that, although it is increasingly popular to use more state-of-the-art, machine-learning-based models, conventional linear models may still play an important role in clinical prediction tasks, if tricky techniques can be well handled (like ridge regression) [37].

Regarding the evaluation of model performance, we should bear in mind that the model was developed from imbalanced data (i.e., the sample size of non-SGA was much larger than that of SGA neonates). In this scenario, the metric of the AUPRC in evaluating the model performance is more intuitive and informative than the commonly used metric of AUROC [31]. For instance, in the case of imbalanced data, the prediction model might show good performance with the measurement of AUROC but the measurement of AUPRC could finally uncover its nature of unsatisfactory performance. However, almost all of the models established for predicting SGA in the imbalanced data had not been assessed its performance with the appropriate measurement indicator of AUPRC [13,45].

Interestingly, maternal height was identified as the most important predictor of SGA in this study population. Our finding was echoed by a study of individual participant data meta-analysis using the data from 12 population-based cohorts in low- and middle-income countries, which also observed that short maternal stature, especially for women < 145 cm, was associated with approximately twice the risk of term SGA and preterm SGA [46]. Our study differed from this meta-analysis in that we treated maternal height as a continuous variable instead of simply categorizing it into groups like < 145 cm, 145 to < 150 cm, 150 to < 155 cm, and ≥ 155 cm. As such, we could avoid the arbitrary categorization and also observe a curvilinear association of maternal height with the risk of SGA. This finding had important clinical implications in prenatal care where much focus has been put on gestational weight gain and pre-pregnancy BMI [47]. Maternal height might be paid equally important attention in prenatal care to reduce the risk of SGA.

### Limitations and strengths

We should interpret the findings of our study cautiously. First, the predictors of SGA did not include the infection factors, but for the pregnant women living in Beijing, China, this may not be a very important factor. This omission in the predictor set could contribute to a performance plateau when the model is applied to diverse populations, as key contextual variables are absent. Second, as in other monocenter studies, the generalizability of our findings to other populations requires further validation. The limited data variability—stemming from a single-center design—may not capture the full spectrum of demographic and environmental factors, potentially exacerbating performance plateaus in external clinical settings and reducing real-world applicability. However, this study mitigated common multi-center data validation challenges through rigorous clinician-led verification, ensuring accuracy in gestational age, birth weight, and SGA diagnosis. However, this study could well overcome the challenges of data validation commonly faced in multi-center studies through the work of careful validation by experienced clinicians, ensuring the accuracy of gestational age, birth weight, and the diagnosis of SGA. Third, the prediction of the high-risk SGA appeared to be underestimated in our calibration analysis of the developed model. These factors could hinder the model’s ability to adapt to complex clinical scenarios, such as varying patient comorbidities or regional healthcare disparities. Nevertheless, our studies also had several strengths in their large sample size, high-quality and clinically validated predictors, a comprehensive type of models including both conventional linear models and complex machine learning models.

### Suggestions for future studies

Conducting SGA prediction studies is crucial in populous countries like China, given the high annual incidence of SGA neonates. The ranking of key SGA predictors varies significantly by country, directly impacting prevention priorities. For example, preventing maternal infections (e.g., malaria) has greater impact in endemic regions, while nutritional interventions are more effective where non-infectious factors(e.g., maternal short height, inadequate gestational weight gain) [26]. This single-center Chinese study primarily involved urban residents. Population differences across countries – including genetics, nutrition, and social environment – may alter predictor weights in models, potentially requiring recalibration of thresholds like maternal height (e.g.,taller Europeans vs. East Asians) [48]. Despite these variations, the biological role of maternal height in SGA remains universal. Our model thus provides a foundational framework; using transfer learning [49] to fine-tune key parameters (such as height thresholds) enables cross-national adaptation. To build a useful prediction model applied in the clinical setting, it is not only important to comprehensively evaluate model performance in terms of discrimination and calibration but also important to consider the feasibility and interpretability of the machine learning model which are crucial factors that clinicians could well adopt the model into their routine clinical practice. There are many cases in which researchers have invested much effort and time into developing a complex prediction model, but regrettably, its translation into real-world clinical practice has been hindered by clinicians’ low acceptance. To bridge this research-utility gap, we think it is crucial to conduct 3 aspects of work: (1) using non-invasive predictors that are also easy to collect; (2) evaluating the performance of developed models as comprehensively as possible (AUROC, AUPRC, calibration); and (3) fully showing the clinical utility of model from different angles in an understandable manner (decision curve analysis and nomogram).

## Conclusions

Our study not only contributes a basic model for the prediction of SGA but also identifies maternal height as an important but previously neglected predictor of SGA. Findings from our study could pave the way for future extended models including biomarkers based on our development of the basic prediction model.

## Supporting information

S1 Fig Nomogram for the prediction of SGA. (TIF)
