# Supplementary figures and images for: Using machine learning to predict the small for gestational age and identify the important predictors: A real-world clinical cohort study in China
Source: PLoS One. 2026 Mar 25;21(3):e0343994. doi: 10.1371/journal.pone.0343994 (PMC13016299; doi:10.1371/journal.pone.0343994)

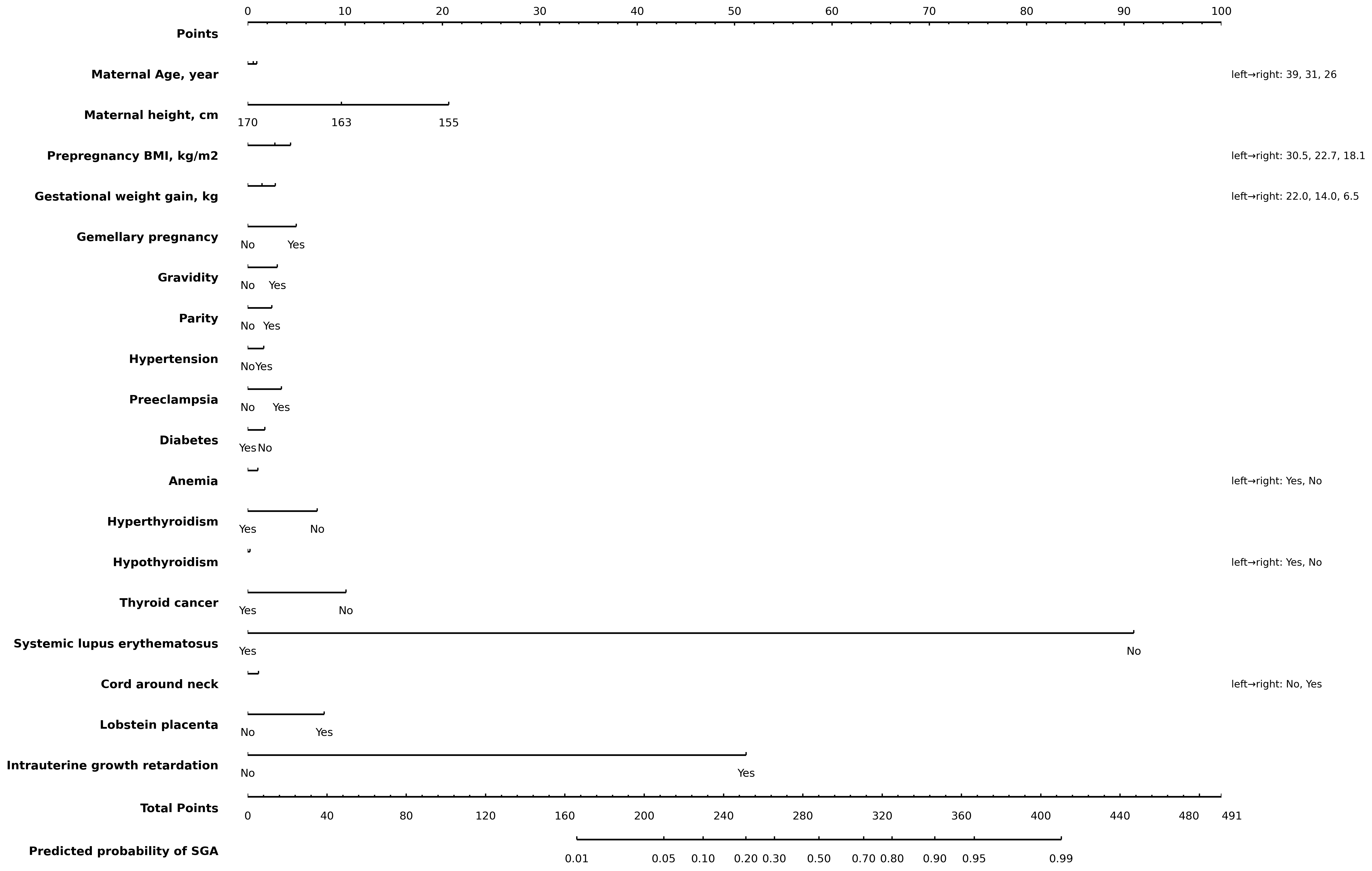

Supplement: S1 Fig — (TIF) [file pone.0343994.s001.tif]
